# *Bacillus amyloliquefaciens* SQR9 induces dendritic cell maturation and enhances the immune response against inactivated avian influenza virus

**DOI:** 10.1038/srep21363

**Published:** 2016-02-19

**Authors:** Lulu Huang, Tao Qin, YinYan Yin, Xue Gao, Jian Lin, Qian Yang, Qinghua Yu

**Affiliations:** 1 Nanjing Agricultural University, Weigang 1, Nanjing, Jiangsu, 210095, PR China

## Abstract

The objective of this study was to evaluate the stimulatory effects of *Bacillus amyloliquefaciens* SQR9 on dendritic cells (DCs) and to verify its ability to enhance the immune response by modulating DC maturation. The results demonstrated that *B. amyloliquefaciens* SQR9 can adhere to the nasal epithelium and be taken up by DCs in the nasal mucosa, thereby inducing DC maturation and resulting in increased CD80, CD86, CD40 and MHCII expression and cytokine secretion. The frequencies of CD4^+^ and CD8^+^ T cells and CD69^+^ memory T cells were increased in spleens after nasal immunization with virus plus *B. amyloliquefaciens* SQR9 compared to immunization with inactivated H9N2 AIV alone. Moreover, the levels of sIgA in the nasal cavity, the trachea, and the lung and the levels of IgG, IgG1, and IgG2a in serum were significantly increased in mice administered WIV plus SQR9 compared to mice administered H9N2 WIV alone. The results of this study demonstrated that *B. amyloliquefaciens* SQR9 can stimulate DC maturation to effectively induce an immune response. In conclusion, an effective immune response may result from the uptake of H9N2 by DCs in the nasal mucosa, thereby stimulating DC maturation and migration to cervical lymph nodes to initiate immune response.

Probiotics have repeatedly been shown to have many beneficial effects within the gut, such as maintaining the microbiota balance, defending against enteropathogen invasion, and enhancing immune responses^1^,^2^. Gram-positive bacteria of the genus *Bacillus* are not only widely applied to plants as biocontrol agents^3^ but are also used in human and animal feed products and as live organisms for human consumption^4^. Killed *Bacillus subtilis* spores and surfactin lipopeptide fungin from *Bacillus amyloliquefaciens* have been chosen as mucosal adjuvants to elicit strong immune responses^5^,^6^. Recombinant *Bacillus subtilis* expressing heterologous antigens has been demonstrated to be effective as an antigen delivery system by inducing effective immune protection against *Clostridium perfringens*^7^ and rotavirus^8^.

*Bacillus amyloliquefaciens* SQR9, which was isolated from soil, displays high similarity to *Bacillus subtilis*. This bacterial strain exhibits good biocontrol characteristics in the cucumber rhizosphere and protects the host plant from pathogen invasion via efficient root colonization^9^. Previous data demonstrated that *B. amyloliquefaciens* SQR9 displays excellent properties for further uses. *Bacillus* can survive in the mucosal tract and directly interact with the mucosal epithelium. However, the details of how *Bacillus* modulates the mucosal environment (i.e., dendritic cells (DCs)) to affect the immune response are unknown.

DCs are ubiquitous cells underlying the mucosa that act as sentinels by monitoring for pathogenic microbes, allergens and pollutants. DCs can recognize microbes via multiple pattern recognition receptors (PRRs), which can activate divergent and polarized adaptive responses^10^. DCs in peripheral tissues are immature and have the capacity to efficiently stimulate T cells. After challenge with antigens, DCs enter the process of maturation and migrate to lymphoid organs^11^. Immature DCs are specialized for antigen capture and processing but weakly stimulate T cells. Only mature DCs displaying high levels of surface expression of major histocompatibility complex class II (MHCII) and costimulatory molecules can efficiently stimulate T cell activation^12^.

In these experiments, mouse bone marrow-derived DCs were isolated and co-cultured with *B. amyloliquefaciens* SQR9. The aim of this study was to investigate the stimulatory activity of *B. amyloliquefaciens* SQR9 on DCs and to detect the immune adjuvant effect of *B. amyloliquefaciens* SQR9 upon intranasal vaccination with inactivated H9N2 avian influenza virus. We hypothesized that *Bacillus could* adhere to the nasal mucosa and influences the maturation of DC, thereby stimulating the mucosal immune response against inactivated virus.

## Results

### SQR9 adhesion to nasal epithelial cells *in vivo*

**Figure 1:**
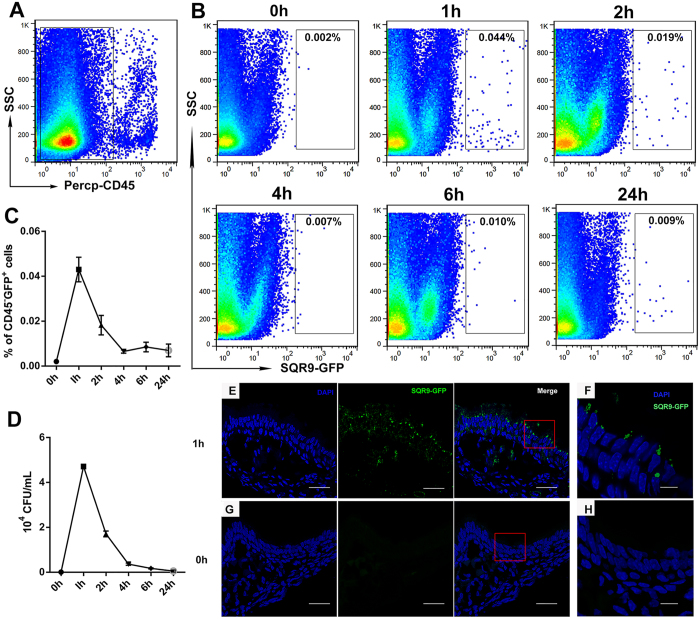
Adhesion of SQR9 to nasal epithelial cells *in vivo*. Mice were intranasally administered PBS or SQR9-GFP (10^7 ^CFU). After 1, 2, 4, 6 or 24 h, the nasal cavities were collected. For FACS analyses, nasal-associated lymphoid tissue was removed from the nasal cavity. The individual cells isolated from the nasal passages were gated for CD45^−^ (**A**) to select non-hematopoietic cells. To evaluate the adhesion or uptake of *B. amyloliquefaciens* SQR9 by epithelial cells, the gated cells were further selected based on GFP^+^ via FACS (**B**,**C**) or the standard plate counting method, which consists of diluting a sample with sterile saline or phosphate buffer diluent until the bacteria are sufficiently diluted to enable accurate counting (**D**). Frozen sections of nasal mucosa treated with SQR9-GFP (**E**,**F**) or PBS (**G**,**H**) were stained with 4′, 6-diamidino-2-phenylindole (DAPI). Error bars mean standard deviation (SD). Bars: 30 μm (**E**,**G**); 10 μm (**F**,**H**).

The adhesion of bacteria to mucosal epithelial cells is crucial for their subsequent uptake by submucosal DCs. Therefore, we performed FACS analysis to explore whether SQR9 can attach to nasal epithelial cells (Fig. 1A,B). The number of CFUs was enumerated using the conventional plate counting technique (Fig. 1D). The number of bacteria that adhered to the epithelium peaked at 1 h and remained detectable at 24 h based on FACS analysis. The numbers of bacteria were verified by CFU counting among CD45^−^GFP^+^ epithelial cells. The adhesion of SQR9 to nasal mucosal epithelial cells was also verified *in vivo* based on confocal microscopy (Fig. 1E,F).

### Uptake of SQR9 by DCs

**Figure 2:**
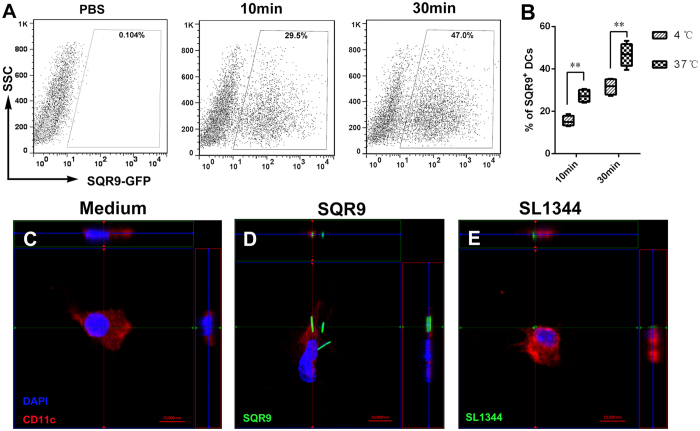
Cellular uptake of SQR9 by dendritic cells (DCs) *in vitro.* DCs were incubated in GFP-labeled *B. amyloliquefaciens* SQR9 (SQR9-GFP) for 10 min or 30 min (**A,B**) and washed twice with PBS, followed by FACS analysis. Confocal microscopy was used to detect DCs (**C**), DCs+SQR9 (**D**) or DCs+SL1344 (**E**). Frozen sections were stained with CD11c (red) and DAPI (blue). The experiments were repeated for three independent experiments. The data are presented as the means ± SD. N = 3. Error bars mean standard deviation (SD). *P < 0.05; **P < 0.01.

DC maturation is crucial for the initiation of downstream immune responses. To identify the impact of SQR9 on innate immune cell activation, we evaluated whether SQR9 affects the uptake of bacteria by DCs. DCs were incubated in the presence or absence of GFP-labeled SQR9 (SQR9-GFP; 10^7^ CFU) for 30 min. Then, the internalized SQR9-GFP was detected by FACS and confocal laser scanning microscopy. Our FACS data showed that SQR9 efficiently enhanced the ability of DCs to take up bacteria (Fig. 2).

### SQR9 promotes DC maturation *in vitro*

**Figure 3:**
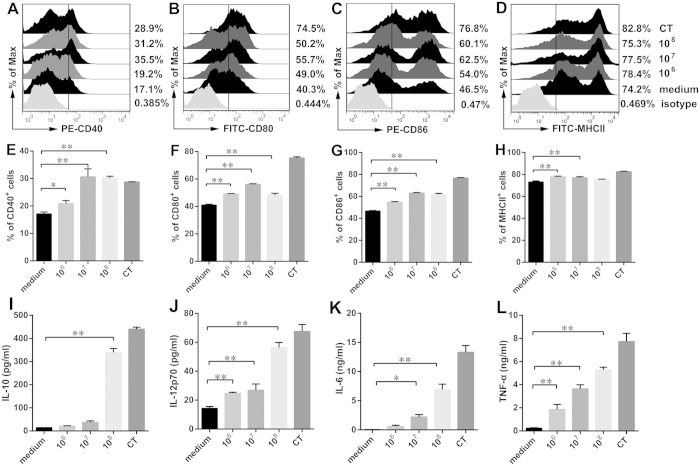
SQR9 enhances DC maturation. DCs were incubated in *B. amyloliquefaciens* SQR9 (10^6^ CFU, 10^7^ CFU or 10^8^ CFU) or cholera toxin (CT, 2 μg/mL). After 24 h, DCs and supernatants were collected to detect DCs phenotypes (**A–H**) and DC-mediated cytokine secretion (**I**–**L**). The expression of CD40 (**A**,**E**), CD80 (**B**,**F**), CD86 (**C**,**G**) and MHC-II (**D**,**H**) by DCs was analyzed by FACS. The secretion of IL-10 (**I**), IL-12p70 (**J**), IL-6 (**K**) and TNF-α (**L**) in culture supernatants was measured by ELISA. The experiments were repeated for three independent experiments. The data are presented as the means ± SD. N = 3. Error bars mean standard deviation (SD). *P < 0.05; **P < 0.01.

The expression of co-stimulatory molecules by DCs after SQR9 uptake was detected by flow cytometric analysis. DCs play a crucial role in the effective induction of adaptive immune responses by increasing their expression of MHC molecules. Therefore, we analyzed the expression of surface markers and cytokines. We confirmed that DCs showed significant upregulation of CD80 (Fig. 3B,F), CD86 (Fig. 3C,G), CD40 (Fig. 3A,E) and MHC class II (MHCII) (Fig. 3D,H) in response to treatment with antigen together with SQR9 compared to treatment with antigen alone. Additionally, cytokines play an important role in T cell differentiation and in cell-mediated and humoral immunity. In this study, the levels of IL-10 (Fig. 3I), IL-12p70 (Fig. 3J), IL-6 (Fig. 3K) and TNF-α (Fig. 3F) were measured in the culture supernatants. As shown in Fig. 3, IL-6, IL-12 and TNF-α production was significantly increased in a dose-dependent manner (*P* < 0.01), leading to an enhanced response to the co-administered antigen. Thus, it is conceivable that SQR9 can activate antigen-presenting cells. Furthermore, our results showed that SQR9 was capable of inducing cytokine production. These findings suggested that SQR9 was capable of enhancing the phenotypic maturation of DCs.

### SQR9 facilitates the H9N2 WIV-induced recruitment of submucosal DCs and antigen capture in cervical lymph nodes (CLNs)

**Figure 4:**
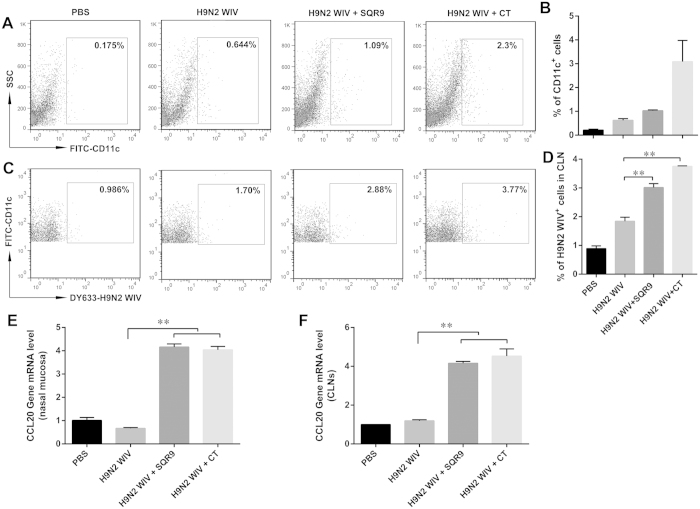
SQR9 recruits DCs and increases the number of H9N2 WIV-loaded DCs in CLNs. (**A–D**) C57BL/6 mice were intranasally administered PBS or DyLight 633-labeled H9N2 WIV (10 μg HA) with or without *B. amyloliquefaciens* SQR9 (10^7 ^CFU) or cholera toxin (CT, 2 μg) for 90 min. Epithelial cells from the nasal mucosa and cervical lymph node (CLN) cells were isolated and analyzed by FACS. (**A**,**B**) FACS analysis of FITC-CD11c^+^ cells from nasal mucosal epithelial cells. (**C**,**D**) FACS analysis of DyLight 633 virus-loaded DCs based on gating for FITC-CD11c^+^ cells from CLNs. (**E**,**F**) C57BL/6 mice were intranasally administered PBS or H9N2 WIV (10 μg HA) with or without *B. amyloliquefaciens* SQR9 (10^7^ CFU) or cholera toxin (CT, 2 μg) for 12 h. (**E**,**F**) Chemokine (C-C Motif) Ligand 20 (CCL20) expression in the nasal mucosa and in CLNs was detected by real-time quantitative PCR. The experiments were repeated for three independent experiments. The data are presented as the means ± SD. N = 3. Error bars mean standard deviation (SD). *P < 0.05; **P < 0.01.

The use of inactivated influenza viruses as vaccine antigens is much less dangerous to nasal epithelial cells than the use of live viruses. We hypothesized that SQR9 enhances virus uptake by submucosal DCs, possibly providing an advantage in terms of vaccination efficiency. We collected CLN cells after nasal instillation of PBS, H9N2 WIV alone, or H9N2 WIV together with SQR9 or cholera toxin (CT). FACS analyses indicated that SQR9 or CT significantly increased the number of H9N2 WIV-loaded DCs in the CLN (Fig. 4C,D). These data combined with the data presented in Fig. 2 suggested that prolonged adhesion of *B. amyloliquefaciens* SQR9 to the nasal mucosa enhanced the activation of innate immunity. Thus, SQR9 administration recruited DCs to submucosal regions (Fig. 4A,B), such as the nasal mucosa, and induced virus uptake, thereby enabling virus-loaded DCs to rapidly migrate to CLNs for antigen presentation. This phenomenon was confirmed via immunofluorescence staining of the nasal mucosa and CLNs followed by confocal microscopy. After treatment with SQR9 or CT, the number of DCs in the nasal mucosa was increased, and the abundance of H9N2 WIV in CLNs was elevated (Figure S2). Moreover, the CCL20 mRNA expression level in the nasal mucosa of group treated with H9N2 WIV plus SQR9 increased 6.2 fold than that of H9N2 WIV alone, while the CCL20 mRNA expression level in CLNs increased 3.4 fold (Fig. 4E,F). The increased number of DCs in the nasal mucosa and in CLNs may be attributed to the elevated mRNA expression of CCL20 in the nasal mucosa and in CLNs upon treatment with SQR9 or CT.

### Specific sIgA levels in the respiratory tract

**Figure 5:**
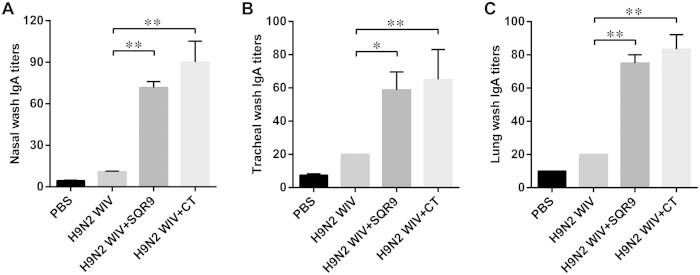
SQR9 assisted H9N2 WIV in enhancing the local immune response after nasal immunization in mice. The antigen-specific mucosal IgA titers in nasal washes (**A**), tracheal washes (**B**), and lung washes (**C**) of immunized mice were detected 2 weeks after the final vaccination. The experiments were repeated for three independent experiments. The data are presented as the means ± SD. N = 3. Error bars mean standard deviation (SD). *P < 0.05; **P < 0.01.

To determine whether immunization enhanced mucosal cell-mediated immune responses, the induction of the local anti-H9N2 WIV-specific sIgA expression levels in the nasal cavity, the trachea and the lungs were measured 28 days after the first immunization. The specific sIgA antibody levels were determined via ELISA (Fig. 5). The mucosal sIgA levels in the nasal cavity (Fig. 5A), tracheal (Fig. 5B) and lung washes (Fig. 5C) were significantly enhanced after intranasal immunization with H9N2 WIV combined with either SQR9 or CT (*P *< 0.01) compared to immunization with H9N2 WIV antigen alone.

### Specific IgG levels in serum

**Figure 6:**
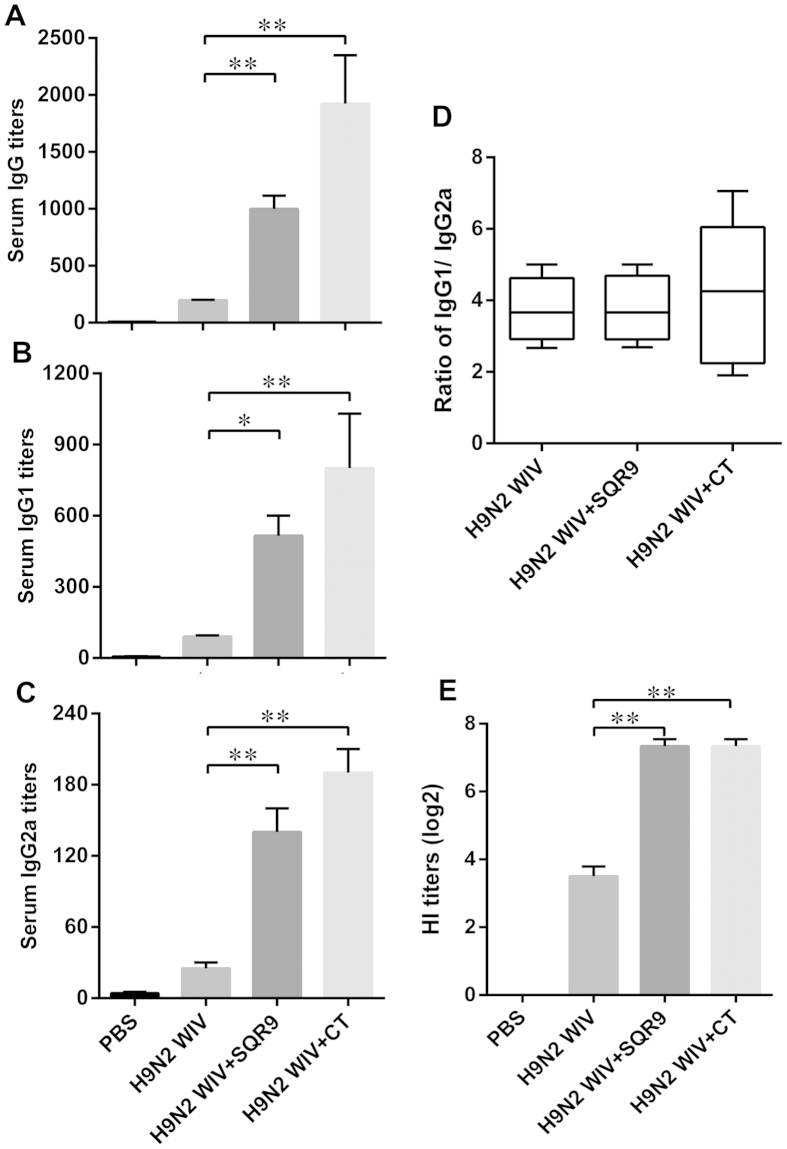
SQR9 assisted H9N2 WIV in enhancing systemic immune responses after nasal immunization in mice. The antigen-specific serum IgG titers (**A**), IgG1 titers (**B**), IgG2a titers (**C**), and hemagglutination inhibition (HI) titers (**D**) in immunized mice were detected 2 weeks after the final vaccination. The experiments were repeated for three independent experiments. The data are presented as the means ± SD. N = 3. Error bars mean standard deviation (SD). *P < 0.05; **P < 0.01.

To determine whether immunization enhanced the systemic immune response, the specific anti-H9N2 avian influenza virus (AIV) IgG expression levels in serum were detected 28 days after the first immunization. The specific IgG levels were determined by ELISA (Fig. 6). To establish the antibody subtypes, we measured the IgG2a and IgG1 antibody levels in each group^13^ and found that the serum antigen-specific IgG (Fig. 6A), IgG1 (Fig. 6B) and IgG2a antibody titers (Fig. 6C) were enhanced by SQR9 or CT when administered together with H9N2 WIV. The levels of these systemic antibodies were substantially higher following induction with H9N2 WIV antigen and either SQR9 or CT than following induction with H9N2 WIV antigen alone (*P *< 0.01). Moreover, the IgG1 subtype was dominant in all immunized mice, and lower titers of the IgG2a subtype were detected in sera from the immunized mice. The addition of SQR9 did not alter the IgG1/IgG2 ratio, which exceeded 1 in both the WIV group and the WIV and SQR9 group. These results suggested that the IgG1 subtypes may play a major role in the efficiency of the immunization of mice with an AIV vaccine together with SQR9. Additionally, we analyzed the hemagglutination inhibition (HI) titer in serum. H9N2 WIV combined with SQR9 also increased the HI titer compared to H9N2 WIV alone (Fig. 6D). Taken together, these results indicated that H9N2 WIV together with SQR9 effectively induced systemic immune responses after intranasal immunization.

### Positive proliferation of spleen lymphocytes after immunization *in vitro*

**Figure 7:**
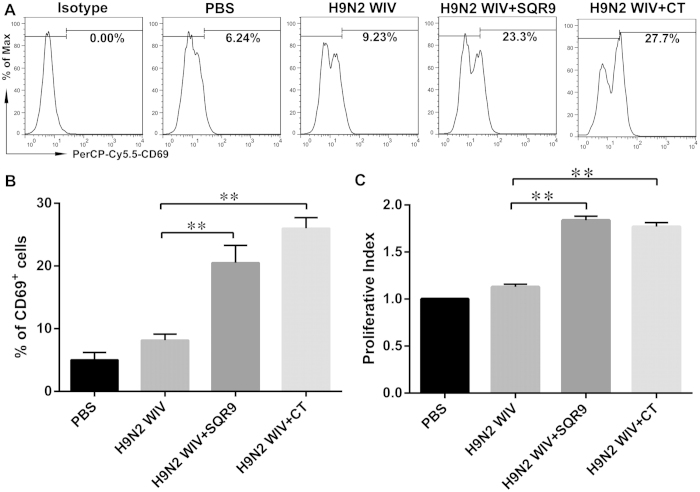
SQR9 assisted H9N2 WIV in enhancing CD69 expression by lymphocytes isolated from the spleen after vaccination. At 28 days after primary immunization, splenic lymphocytes were isolated from immunized mice and re-stimulated with H9N2 WIV *in vitro*. Splenocyte activation was assessed based on CD69 expression via FACS analysis. The proliferative index of the spleen was analyzed using the Cell Counting Kit-8 (CCK-8) assay. The experiments were repeated for three independent experiments. The data are presented as the means ± SD. N = 3. Error bars mean standard deviation (SD). *P < 0.05; **P < 0.01.

**Figure 8:**
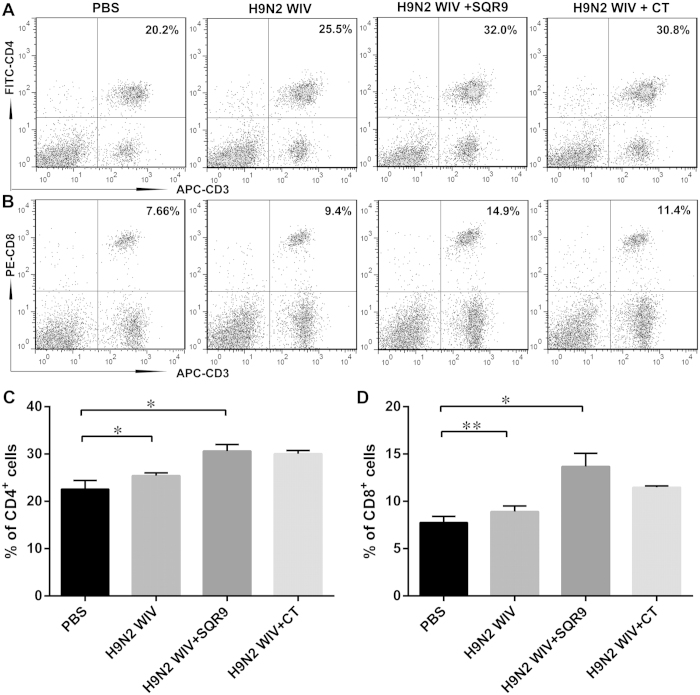
SQR9 assisted H9N2 WIV in enhancing the percentages of CD3^+^CD4^+^ and CD3^+^CD8^+^ T cells. At 28 days after primary immunization, the percentages of CD3^+^CD4^+^ (**A**,**C**) and CD3^+^CD8^+^ (**B**,**D**) splenic T cells from the immunized mice were analyzed by FACS. The experiments were repeated for three independent experiments. The data are presented as the means ± SD. N = 3. Error bars mean standard deviation (SD). *P < 0.05; **P < 0.01.

Splenocytes were isolated from immunized mice and re-stimulated with H9N2 WIV *in vitro*. We found that CD69 expression (Fig. 7A,B) and the proliferative index (Fig. 7C) were markedly increased in the groups immunized with H9N2 WIV and either SQR9 or CT compared with the group immunized with H9N2 WIV alone. To investigate the mechanism underlying the adjuvant effect of SQR9, we investigated cell proliferation. *B. amyloliquefaciens* SQR9 together with the antigen induced a significant increase in the percentages of CD3^+^CD4^+^ T cells (Fig. 8A,C) and CD3^+^CD8^+^ T cells (Fig. 8B,D) compared to the antigen alone. These results indicated that nasal immunization with H9N2 WIV together with SQR9 effectively induced systemic and local immune responses in mice.

## Discussion

The respiratory tract is the main gateway for AIV invasion and infection. Intranasal immunization can induce efficient local immune responses in the mucosa of the respiratory tract that can prevent respiratory tract infection with AIV. However, the natural mucosal barrier is the key impediment to the capture and delivery of inactivated AIV by DCs; therefore, this barrier affects the absorption efficiency of intranasal inoculation. In this study, we found that *B. amyloliquefaciens* SQR9 adheres to the mouse nasal epithelium, as indicated by the detection of CD45^−^GFP^+^ cells during the first 24 h after treatment; this adhesion enhanced the frequency of DCs underneath the nasal mucosa and contributed to antigen capture. We found that bone marrow-derived DCs can capture *B. amyloliquefaciens* SQR9 and that antigen uptake by DCs increased with increasing culture time and temperature. This phenomenon was verified *in vitro* via confocal microscopy. After phagocytosis of *B. amyloliquefaciens* SQR9, the DCs matured and exhibited increased expression of CD40, CD80, CD86 and MHCII.

Cytokines induce varying biological effects on different cell types during immune processes. IL-10 promotes the differentiation of naive CD4^+^ T cells into Th2 cells^14^, and IL-12 enhances both CD8^+^ T cell-mediated and non-specific cell-mediated immune responses^15^. The stimulatory effect of *B. amyloliquefaciens* SQR9 on DC maturation was confirmed by the increase in the DC-mediated secretion of cytokines, including IL-6, TNF-α, IL-10 and IL-12p70. TNF-α is a rapid proinflammatory cytokine that can stimulate DC maturation^16^,^17^. Therefore, we propose that *B. amyloliquefaciens* SQR9 might induce cell-mediated immune responses.

The data from our *in vitro* assay showed that *B. amyloliquefaciens* SQR9 stimulated the maturation of DCs, which play vital roles in innate immune responses and subsequent adaptive immunity^18^. To investigate whether *B. amyloliquefaciens* SQR9 can stimulate DCs in the nasal mucosa and effectively induce an immune response against an inactivated AIV, we intranasally administered mouse H9N2 WIV together with *B. amyloliquefaciens* SQR9. The stimulatory effect of this treatment on T cells was observed in splenic lymphocytes isolated from mice 28 days after primary nasal vaccination with H9N2 WIV and *B. amyloliquefaciens* SQR9. The stimulation of T cell proliferation by *B. amyloliquefaciens* SQR9 was consistent with that by CT based on CCK-8 assays. CT has widely been used as an effective adjuvant and has been shown to induce both mucosal and systemic immune responses^19^. Furthermore, as an adjuvant, *B. amyloliquefaciens* SQR9 increased the number of CD3^+^CD8^+^ T cells and CD3^+^CD4^+^ T cells in the spleen compared to CT. We also found that primary immunization with H9N2 WIV together with *B. amyloliquefaciens* SQR9 increased the number of CD69^+^ cells in the spleen after re-stimulation with H9N2 WIV compared to primary immunization with H9N2 WIV alone. These results indicated that nasal immunization with H9N2 WIV effectively induced not only CD3^+^CD4^+^ and CD3^+^CD8^+^ T cell proliferation but also immunological memory in the spleen to prevent the next AIV attack.

In addition to the cellular immune response, humoral immunity also plays key roles in defending against pathogen invasion and infection. Other researchers have confirmed that mucosal sIgA is the major mediator of protection from nasal challenge with influenza virus that has invaded the mucosa^20^. In this study, high IgA antibody levels in respiratory tract tissues (nasal, trachea and lung) were detected after intranasal immunization with H9N2 WIV together with *B. amyloliquefaciens* SQR9. This increased expression of sIgA in the respiratory tract was consistent with the enhanced secretion of IL-6, which is capable of inducing B cell proliferation and promoting IgA secretion^21^. The serum antibody IgG is also important for protecting against pathogen invasion. Nasal immunization with H9N2 WIV and *B. amyloliquefaciens* SQR9 significantly increased the IgG levels, and this observation is consistent with the increased expression of neutralizing antibodies, as represented by the HI titer. Moreover, IgG subtypes (IgG2a and IgG1) were investigated to address the influence of *B. amyloliquefaciens* SQR9 on the balance between Th1- and Th2-type immune responses. IgG1 has been reported to be an indicator of a Th2-type immune response, whereas production of IgG2a-type antibodies reflects a Th1-type immune response^22^. The expression levels of IgG1 and IgG2a in serum were significantly increased, and the IgG1 titer was greater than the IgG2a titer in serum. However, the addition of SQR9 did not alter the IgG1/IgG2 ratio. The IgG1/IgG2a ratio was >1 in the H9N2 WIV group and the H9N2 WIV and SQR9 group; this result indicated that H9N2 WIV alone or together with *B. amyloliquefaciens* SQR9 induces a primarily Th2-type antibody response^23^. Thus, *B. amyloliquefaciens* SQR9 was capable of enhancing the humoral immune response at the both local nasal mucosal and the systemic levels.

Antigen uptake by nasal submucosal DCs is the first critical step for subsequent adaptive immunity. H9N2 WIV can be taken up by DCs in the nasal mucosa, and these cells can migrate to a CLN. These events were enhanced by the nasal administration of H9N2 WIV together with *B. amyloliquefaciens* SQR9, implying that *B. amyloliquefaciens* SQR9 can induce a more effective immune response against AIV. C-C ligand chemokines (such as CCL20) are key factors in DC migration. The continuous binding of *B. amyloliquefaciens* SQR9 to the nasal epithelium could stimulate the epithelium to secrete more chemokines, which, in turn, provide DCs with more opportunities to take up luminal AIV and deliver the virus to T cells. High levels of CCL20 mRNA expression in the nasal mucosa and in CLNs were detected in the H9N2 WIV and SQR9 group. This finding could explain how *B. amyloliquefaciens* SQR9 effectively induced mucosal and systemic immune responses.

In conclusion, *B. amyloliquefaciens* SQR9 can bind to the epithelium and stimulate DC-mediated transepithelial antigen uptake and DC maturation. Moreover, *B. amyloliquefaciens* SQR9 elicited robust local mucosal and systemic immune responses after intranasal immunization, and these enhanced effects could be beneficial for the prevention of an H9N2 influenza pandemic. Bacillus has been used in food fermentation for centuries^6^, and *B. amyloliquefaciens* SQR9 did not affect the mouse body weight (Figure S1). Therefore, *B. amyloliquefaciens* SQR9 is a promising adjuvant for modulating DC maturation and DC-mediated antigen uptake to induce strong immune responses against an invading AIV.

## Methods

### Ethics statement

This study was approved by the Ethics Committee for Animal Experimentation of Nanjing Agricultural University. All animal care and use procedures were conducted in strict accordance with the Animal Research Committee guidelines of the College of Veterinary Medicine at Nanjing Agricultural University.

### Vaccine preparation

The influenza A/Duck/NanJing/01/1999 H9N2 virus was generously provided by the Jiangsu Academy of Agricultural Sciences^24^. The virus was purified using a discontinuous sucrose density gradient. Heat-inactivated viruses were prepared via incubation at 56 °C for 0.5 h to achieve a complete loss of infectivity. The purified virus concentrations were measured using the BCA protein assay kit (Thermo Fisher, MA, USA; the HA concentration was approximately 35% of the total protein concentration)^25^. The *B. amyloliquefaciens* SQR9 strain was kindly supplied by Professor Ruifu Zhang of Nanjing Agricultural University^9^,^26^.

### SQR9 adherence assay

BALB/c mice were intranasally administered 10 μL of SQR9-GFP (10^7 ^CFU) or 0.01 M PBS into each nostril. After 1, 2, 4, 6 or 24 h, the mice were anesthetized and sacrificed. The noses were collected after removing the lower jaw together with other excess tissues. For FACS analysis, epithelial cells were isolated from the nasal passage^27^ and stained with CD45-PerCP. The number of CFUs was estimated using the conventional plate counting technique. The nasal mucosa and CLNs were collected for the detection of SQR9 adhesion via confocal microscopy.

### Uptake activity, phenotype and cytokine secretion of DCs upon stimulation by *B. amyloliquefaciens* SQR9

DCs were isolated and cultured using our previously described method^28^. Briefly, bone marrow was obtained from femurs and tibias of wild-type C57BL/6 mice and treated with red blood cell lysing buffer (Beyotime, China). The bone marrow cells were differentiated into DCs by resuspending the cells in complete medium (RPMI 1640 medium supplemented with 10% FBS, 1% penicillin/streptomycin, 10 ng/mL GM-CSF and 10 ng/mL IL-4; PeproTech, USA) and plated at a density of 1 × 10^6 ^cells/ml in six-well culture plates (Corning, USA). Non-adherent granulocytes were removed by discarding the culture medium after 60 h in culture. After 6 days in culture, non-adherent and loosely adherent cells were harvested and centrifuged to remove debris and dead cells. Then, the cells were transferred to 6-well plates and cultured overnight in complete medium.

Next, to explore the uptake of SQR9 by DCs, DCs (5 × 10^5 ^cells) were cultured in serum-free medium and incubated in SQR9-GFP (10^7 ^CFU) for 10 or 30 min at 37 °C. Then, the DCs were washed twice with PBS at 4 °C and analyzed via FACS. Additionally, DCs were stimulated with CT (2 μg/mL) and 1, 10, or 100 MOI of SQR9 for 24 h. The cells were stained with FITC-CD80, PE-CD86, PE-CD40 or FITC-MHCII (eBioscience, USA) for 30 min at 4 °C. The stained cells were analyzed via FACS (FACSCalibur, BD, USA).

The concentrations of IL-10, IL-12p70, IL-6 and TNF-α in the culture supernatants were measured via ELISA using commercial immunoassay kits (eBioscience, CA, USA) according to the manufacturer’s instructions.

### Nasal cavity perfusion experiment and tissue collection

C57BL/6 mice were intranasally administered DyLight 633-labeled H9N2 WIV (10 μg HA) with or without 10 μL of *B. amyloliquefaciens* SQR9 (10^7 ^CFU) or PBS into each nostril. After 90 min, nasal mucosal tissue and CLNs were collected for the detection of CCL20 mRNA expression and immunofluorescence to determine the distribution of DCs. Moreover, individual cells were obtained from the nasal epithelial cells and CLNs by filtering the samples through a 100-μm cell strainer. Then, the isolated cells were stained with FITC-CD11c (1:500, eBioscience, USA) and analyzed via FACS.

### Mice and immunization schedule

Six-week-old BALB/c mice were housed in a specific pathogen-free environment in our animal facility for at least 1 week prior to use. Mice were randomly divided into 4 groups of 40 mice in each group. The groups of mice were intranasally immunized at 0, 7 and 14 days with PBS, H9N2 WIV (containing 10 μg of HA), H9N2 WIV together with SQR9 (10^7 ^CFU), or H9N2 WIV together with CT (2 μg).

### Sample collection

Two weeks after the final immunization, blood samples were collected from 10 mice in each group. Serum was collected after centrifugation and was stored at −70 °C until the detection of anti-AIV-specific total IgG, IgG1 and IgG2a. Then, the mice were euthanized, and their spleens were extracted from the abdominal cavities. Nasal cavity, trachea and lung lavage fluid were washed with 0.5 mL, 0.2 mL or 0.5 mL of sterile PBS, respectively, to collect mucosal suspensions. The suspensions were centrifuged at 5,000 × g for 10 min, and the supernatant was collected and stored at −70 °C.

### ELISA for IgA and IgG

The levels of antigen-specific serum antibodies (total IgG, IgG1 and IgG2a) and of sIgA antibodies in mucosal washes (nasal, tracheal and lung washes) were measured via indirect ELISA as described previously. The ELISA plates were coated overnight at 4 °C with H9N2 WIV and then blocked by incubation in 1% (w/v) BSA in PBS containing 0.05% Tween (PBST) for 1 h at 37 °C. Thereafter, the plates were washed 5 times with PBST (0.01 M, pH 7.4). Two-fold serial dilutions of serum or lavage fluid samples from mice were applied to the plates and incubated for 1.5 h at 37 °C. After washing 5 times with PBST (0.01 M, pH 7.4), the plates were incubated in HRP-conjugated anti-mouse IgG (total IgG, IgG1 or IgG2a) (Santa Cruz, CA) or IgA antibodies (Southern Biotech, Birmingham, AL, USA) for 1 h. The plates were washed 5 times and incubated in 3,3′, 5,5′- tetramethylbenzidine (TMB). After 20 min, the reaction was stopped using sulfuric acid, and the absorbance was measured at 450 nm using a microplate reader. The results were expressed as the ratio of OD_450_ produced by the serum or mucosal wash samples relative to the produced by the negative control serum or mucosal wash sample (P/N). Samples with an OD_450_ ratio higher than 2.1 were considered to be positive. The titer was expressed as the highest dilution of antibody that produced a P/N ratio ≥2.1.


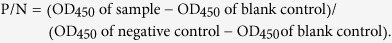


### HI assay

The HI titer for antibodies against the H9N2 strain was determined according to a previously described procedure^29^. The neutralization activity of serum antibodies against the H9N2 strain was measured via the HI assay^30^. Briefly, 50 μL of PBS was added to each well of 96-well plates; then, 50 μL of serum was transferred in and serially diluted 2-fold in PBS. Four hemagglutination units of influenza A/Duck/NanJing/01/1999 were added, and the mixture was incubated for 30 min at room temperature. After the addition of 50 μL of 1% chicken erythrocyte suspension, the plates were incubated for 30 min at room temperature. The highest dilution capable of preventing hemagglutination was scored as the HI titer.

### Stimulation of splenic lymphocytes

Splenic lymphocytes were isolated from immunized mice and stained with APC-CD3, FITC-CD4 and PE-CD8 (eBioscience, USA) at 4 °C for 30 min to identify T cell subtypes via FACS (FACSCalibur, BD, USA). Alternatively, splenic lymphocytes were cultured at a density of 5 × 10^5 ^cells/500 μL and re-stimulated with H9N2 WIV (10 μg/mL) *in vitro* at 37 °C for 72 h.

CD69 activation was assessed via FACS. Lymphocytes were cultured in 96-well culture plates at a density of 10^5 ^cells/100 μL and re-stimulated with H9N2 WIV (10 μg/mL) *in vitro* at 37 °C for 72 h. Proliferative responses were detected using the CCK-8 assay kit according to the manufacturer’s instructions (Beyotime, China). Cells in 96-well plates were incubated in 11 μL of CCK-8 solution for 2 h at 37 °C. The absorbance of each well at 450 nm was quantified using an automated ELISA plate reader. The stimulation index (SI) was calculated using the following formula:


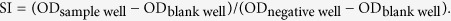


### Statistical analysis

The results were expressed as the means ± standard deviation (SD). Statistical significance was determined using Student’s t test; a value of *P *< 0.05 was considered statistically significant.

## Additional Information

**How to cite this article**: Huang, L. *et al. Bacillus amyloliquefaciens* SQR9 induces dendritic cell maturation and enhances the immune response against inactivated avian influenza virus. *Sci. Rep.* **6**, 21363; doi: 10.1038/srep21363 (2016).

## Supplementary Material

Supplementary Information
