# Supplementary material for: Bacillus amyloliquefaciens SQR9 induces dendritic cell maturation and enhances the immune response against inactivated avian influenza virus
Source: Sci Rep. 2016 Feb 19;6:21363. doi: 10.1038/srep21363 (PMC4759567; doi:10.1038/srep21363)
Supplement: Supplementary Information [file srep21363-s1.pdf]

Bacillus amyloliquefaciens SQR9 induces dendritic cell maturation and enhances the immune response against inactivated avian influenza virus

Lulu Huang, Tao Qin, YinYan Yin, Xue Gao, Jian Lin, Qian Yang, Qinghua Yu*

Nanjing Agricultural University, Weigang 1, Nanjing, Jiangsu, 210095, PR China

*Corresponding author. Tel: +8602584395817; E-mail: yuqinghua1981@163.com (Qinghua Yu).

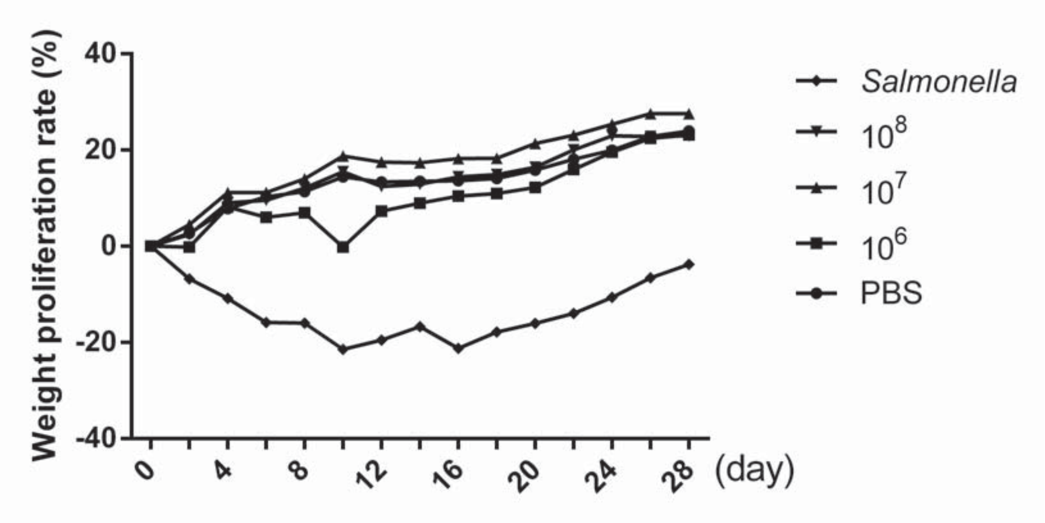

Weight proliferation rate (%)
40
20
0
-20
-40
0
4
8
12
16
20
24
28
(day)
Salmonella
10^8
10^7
10^6
PBS

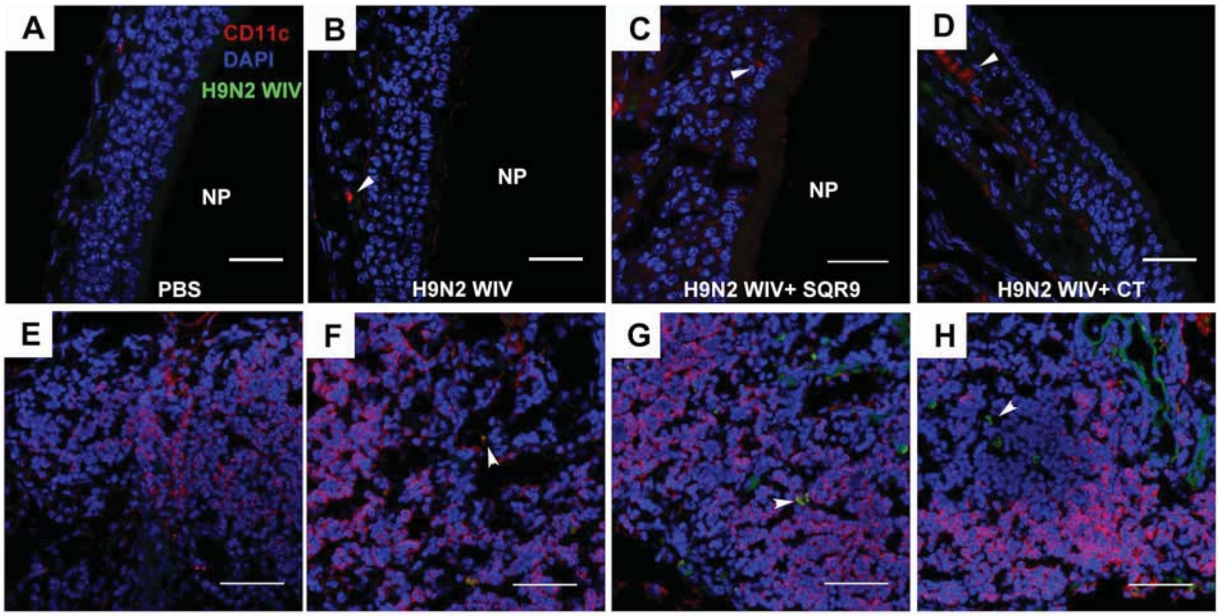
A
CD11c
DAPI
H9N2 WIV
NP
PBS
B
NP
H9N2 WIV
C
NP
H9N2 WIV+ SQR9
D
H9N2 WIV+ CT
E
F
G
H

Figure S1 Rate of body weight changes in the immunized mice. Mice were randomly divided into 4 groups of 40 mice each and challenged with PBS, *B. amyloliquefaciens* SQR9 (10^6, 10^7, or 10^8 CFU) or *S. typhimurium* SL1344 (10^7 CFU) at 0, 7 and 14 days. Then, the mice were monitored every two days to determine the rates of survival and body weight changes. N=3. $^{**}P < 0.01$ vs. the *S. typhimurium* group.

Figure S2 DCs recruitment to nasal epithelial cells and cervical lymph nodes by SQR9. C57BL/6 mice were intranasally administered PBS or SQR9 (10^7 CFU) with or without H9N2 WIV (10 μg HA, green) or cholera toxin (CT, 2 μg) for 90 min. The nasal passage (A-D) and cervical lymph nodes (E-H) were isolated and processed for confocal laser scanning microscopy. Cryosections were immunostained for CD11c (red) and stained with DAPI (blue). Bars: 40 μm.
